# Editorial: Supramolecular cancer therapeutic biomaterials-volume II

**DOI:** 10.3389/fchem.2025.1574334

**Published:** 2025-03-05

**Authors:** Yong Yao, Guifei Huo, Ruibo Zhao

**Affiliations:** ^1^ School of Chemistry and Chemical Engineering, Nantong University, Nantong, China; ^2^ Department of Chemistry, National University of Singapore, Singapore, Singapore; ^3^ Department of Materials, Imperial College London, London, United Kingdom

**Keywords:** supramolecular chemistry, metal-organic framework, dynamic interactions, biomaterials, cell imaging

Supramolecular cancer therapeutic biomaterials are a promising material for cancer treatment (Lv et al., 2022; Zhang H. et al., 2022; Goor et al., 2017). These biomaterials are designed and prepared based on supramolecular chemistry principles (Zhang X. et al., 2022; Chen et al., 2023). Through intermolecular non-covalent forces, they form assemblies with specific properties and complex structures (Klawa et al., 2024; Wang J. et al., 2022). These materials have several advantages in the process of cancer treatment. First, they can be precisely designed to specifically target cancer cells and reduce damage to healthy tissues (Tang et al., 2024; Webber et al., 2016; Wang et al., 2022b). By combining targeting motifs or using stimulus-responsive elements, they can be selectively enriched at the tumor site (Zhang et al., 2023; Hazarika and Singh, 2023; Brito et al., 2021.).

Supramolecular cancer therapeutic biomaterials can also be designed to load and release anticancer drugs in a controllable manner (Mann et al., 2018; Wang et al., 2022c). This enables continuous drug delivery, improves therapeutic effects, and minimizes toxic side effects at the same time (Yang et al., 2022; Wu et al., 2018). In addition, some of these biomaterials can be triggered by external stimuli such as light, heat, or magnetic fields, thereby enabling targeted activation of therapeutic agents (Zhou et al., 2024; Guo et al., 2020). Interestingly, supramolecular biomaterials can exhibit unique properties such as self-assembly, reversible interactions, and adjustable mechanical properties (Cui et al., 2019; Shi et al., 2022). This flexibility allows the design of multifunctional platforms that can combine different treatment methods such as chemotherapy, photothermal therapy, photodynamic therapy, and multimodal synergistic therapy (Wen et al., 2024; Deiser et al., 2023). In this context, we organized the Research Topic of “Supramolecular Cancer Therapeutic Biomaterials” in 2022 and published 8 important articles, showing the latest research results in this field (Yao et al., 2023). Due to the importance and popularity of this Research Topic, we now organize the second volume on this topic. Here, we briefly introduce the research work of this new topic.

Hepatocellular carcinoma (HCC) has the fourth highest death rate among all cancer types worldwide. Programmed cell death (PCD) is a key biological mechanism for controlling cancer progression, tumor expansion and metastasis. In addition, the Tumor microenvironment (TME) is critical in influencing Overall survival (OS) and immune response to immunotherapy interventions. From a multi-omics perspective, the combination of PCD and TME helps predict survival and response to immunotherapy in patients with liver cancer. Liu et al. analyzed changes in the PCD and TME classifiers used to classify liver cancer patients into two subgroups (high PVD-low TME and low PVD-high TME). Next, they compared Tumor somatic mutation (TMB), immunotherapy response, and functional annotation between the two groups. Finally, Western blot (WB) was performed. Immunohistochemistry (IHC) was assessed on the Human Protein Map (HPA). In the PCD-TME classifier, 23 PCD-related genes and three immune cell types were identified. Using this model, patients’ prognosis and response to treatment can be accurately predicted. The results of this study provide a new tool for the clinical management of patients with hepatocellular carcinoma and help develop accurate treatment strategies for these patients.

Fluorescence imaging in the near-infrared-II region has the advantages of centimeter-scale tissue penetration and micron-scale spatial resolution, which has sparked interest in visualizing the lymphatic system. Jin et al. prepared HA@ICG nanoparticles based on the NIR-II fluorescence characteristics of ICG, which inhibited the π-π stacking between ICG molecules, had an ideal particle size and surface modification, good imaging duration and resolution, and could assess local microcirculation. *In vitro* and *in vivo* studies demonstrated that it had excellent photostability, biosafety and visualization ability of the lymphatic system, and was expected to be used in the clinic (Zhang et al.). However, further research was needed in the validation of disease models and the imaging of deep-seated lymphatic tissues.

In a research paper, Prof. Wang and coworkers proposed a metal-organic framework (MOF)-based nano-platform for mitochondrial-targeted CO gas therapy and drug combination therapy. They designed a thiol-functionalized MOF (UiO-66-SH) and combined it with the drug resveratrol (RES) to form a UiO@FeCO@RES nano-platform. The platform is capable of triggering decomposition by ATP within tumor cells, releasing RES and generating CO gas, achieving synergistic anti-cancer effects by inhibiting ATPase and disrupting mitochondrial function. Experimental results show that the nano-platform can effectively target mitochondria and release CO gas in response to ROS, significantly enhancing the killing effect on cancer cells (Wang et al.). This strategy of combining gas therapy and drug therapy provides new ideas for cancer treatment.

In a mini-review paper, Fukuhara et al. focuses on the application progress of dynamic and stimuli-responsive supramolecular chemosensors in cancer detection, with particular attention to the external stimulus of solution-state hydrostatic pressure and its role in biological systems. Focusing on the mechanical force of hydrostatic pressure, it was found in HeLa cells that it regulates the influx of Ca^2+^ through the piezo ion channel, mainly affecting the intracellular pathway, and the cells have a dynamic mechanism to restore the original state of the channel. These findings suggest the importance of dynamic and stimuli-responsive supramolecular chemosensors in biological systems (Matsumoto et al.). The different pressure-responsive characteristics of peptide scaffolds can provide ideas for the development of new supramolecular imaging reagents and promote the development of future mechanobiology.

In summary, we highly value the endeavors, understandings, and outlooks of every contributor to the domain of supramolecular cancer therapeutic biomaterials. We sincerely hope that this issue can offer a perspective on applying supramolecular chemistry to address particular biomedical issues and encourage in - depth research within this field.
